# Supplementary material for: Effect of Opioid Receptor Activation and Blockage on the Progression and Response to Treatment of Head and Neck Squamous Cell Carcinoma
Source: J Clin Med. 2023 Feb 6;12(4):1277. doi: 10.3390/jcm12041277 (PMC9967316; doi:10.3390/jcm12041277)
Supplement: Supplementary file 1 [file jcm-12-01277-s001.zip › jcm-2172471-supplementary.pdf]

Journal of
Clinical Medicine

MDPI

Supplementary Materials

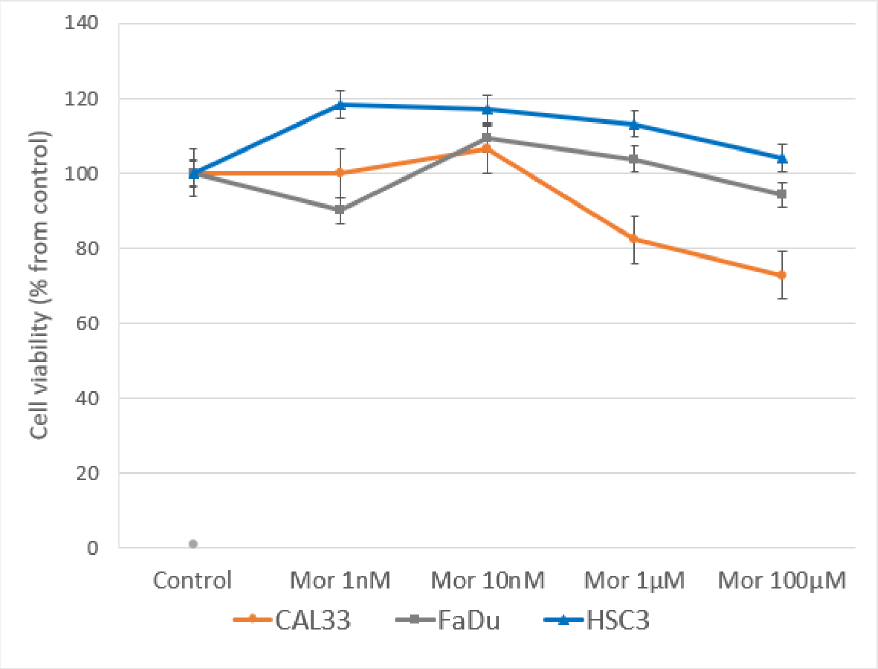

Figure S1. Cell proliferation and toxic doses were assessed in a dose dependent fashion for Morphine, with gradually increasing dosages: 1nM, 10nM, 1µM and 100µM.

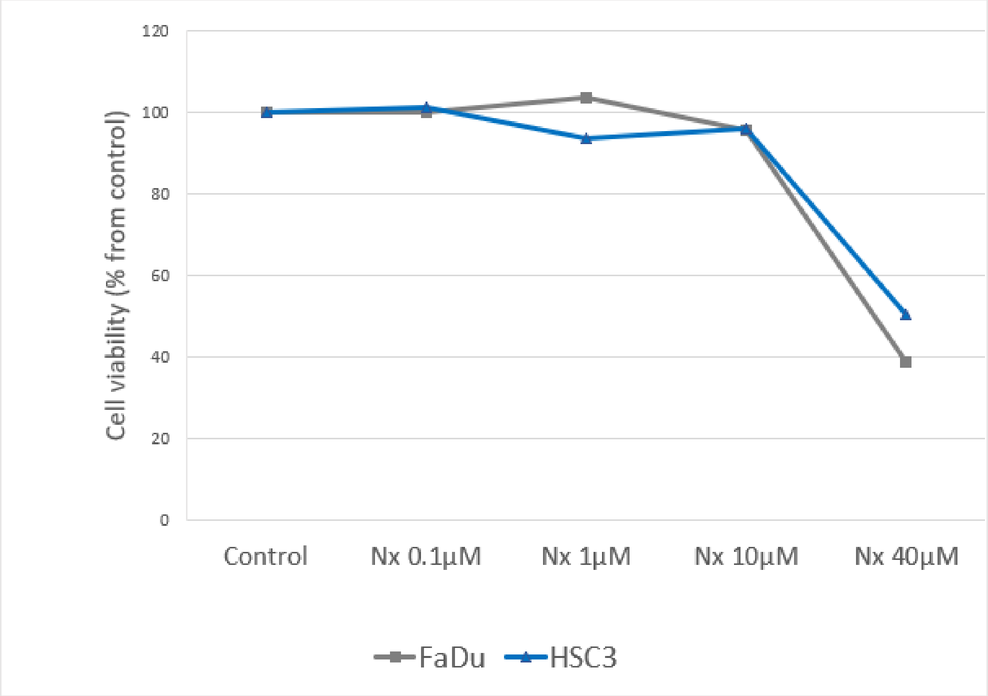

Figure S2. Cell proliferation and toxic doses were assessed in a dose dependent fashion for Naloxone, with gradually increasing dosages: 0.1µM, 1µM, 10µM, 20µM and 40µM. Toxic effect of Naloxone was observed in concentrations over 20µM.

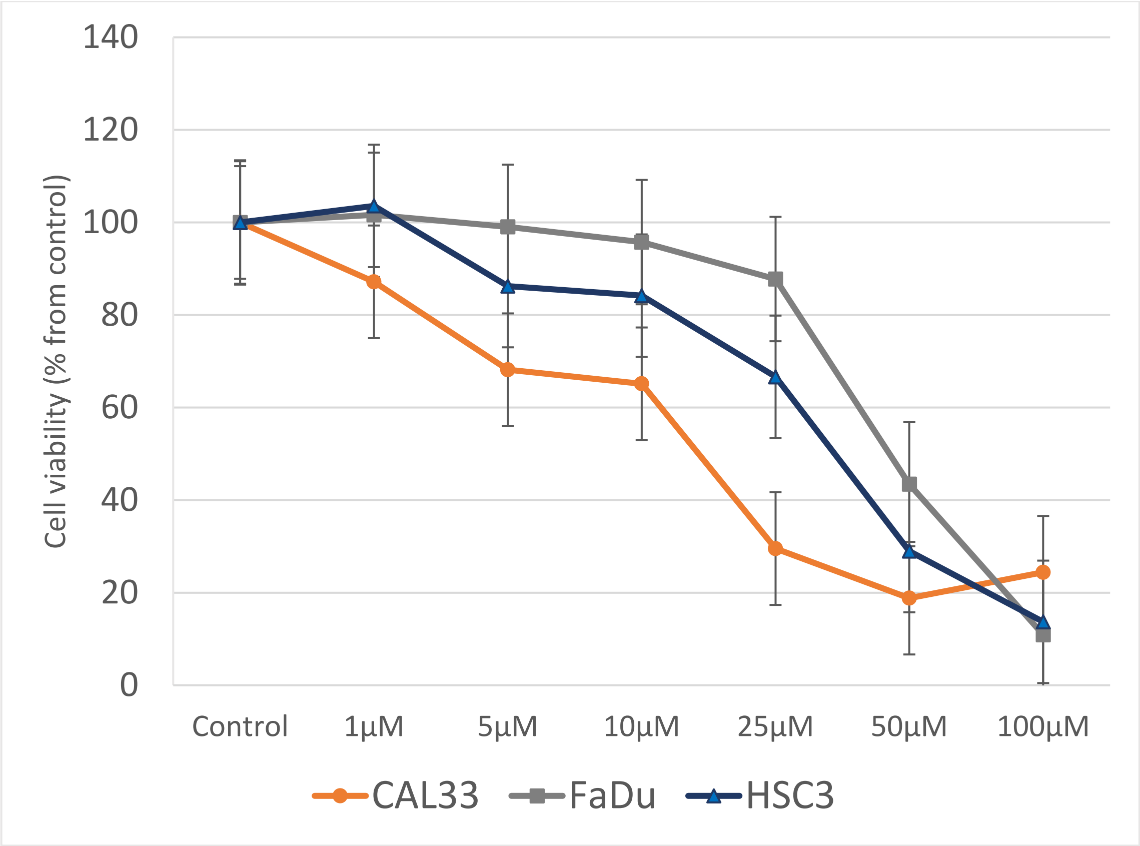

Figure S3. Cell viability of Cal33, FaDu, HSC3 cancer cell lines incubated with gradually increasing dosage of Cisplatin 1µM, 5µM, 10µM, 25µM, 50 µM and 100µM. Cisplatin induced reduction in cell viability in a dose dependent manner.

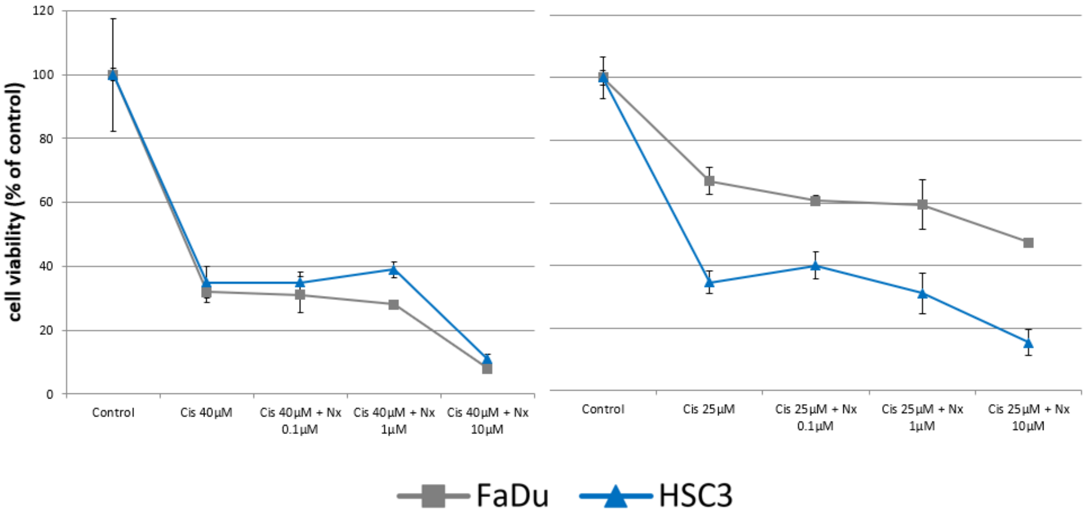

Figure S4. Cell viability of FaDu, HSC3 cancer cell lines incubated with various concentrations of Cisplatin in combination with increasing doses of Naloxone 0.1µM, 1µM, 10µM. Naloxone with Cisplatin induced reduction in cell viability in a dose dependent manner.
